# Exogenous melatonin treatment affects ascorbic acid metabolism in postharvest ‘Jinyan’ kiwifruit

**DOI:** 10.3389/fnut.2022.1081476

**Published:** 2022-12-02

**Authors:** Zhenyu Luo, Jieru Zhang, Miaolian Xiang, Jiaoke Zeng, Jinyin Chen, Ming Chen

**Affiliations:** ^1^College of Agronomy, Jiangxi Agricultural University, Nanchang, China; ^2^Jiangxi Key Laboratory for Postharvest Technology and Non-destructive Testing of Fruits & Vegetables, Nanchang, China

**Keywords:** kiwifruit, melatonin, ascorbic acid, gene expression, metabolism

## Abstract

Ascorbic acid (AsA) is an important nutritious substance in fruits, and it also can maintain the biological activity of fruits during storage. This research investigated the effect of exogenous melatonin (MT) on AsA metabolism in postharvest kiwifruit. Our results indicated that exogenous MT delayed the decrease of fruit firmness and titratable acid (TA), inhibited the increase of soluble solids content (SSC), reduced the respiration rate and ethylene production, and maintained a higher AsA content in kiwifruit during storage. The high expression of L-galactose pathway key genes in the early storage and regeneration genes in the later storage maintained the AsA content in postharvest kiwifruit. MT treatment enhanced the expression levels of AsA biosynthesis (*AcGME2*, *AcGalDH*, and *AcGalLDH*) and regeneration (*AcGR*, *AcDHAR*, and *AcMDHAR1*) genes. Meanwhile, the expression of the degradation gene *AcAO* was inhibited in MT-treated kiwifruits.

## Introduction

Kiwifruit (*Actinidia* spp.) is famous for its high content of ascorbic acid (AsA), which is considered to be one of its key nutritional characteristics. During the postharvest process, AsA content decreases with the ripening and senescence of kiwifruit. As a rich small molecule substance, AsA is closely related to the stress resistance, antioxidant capacity, cell division and photosynthesis of plants. Due to gene mutations encoding enzymes in AsA biosynthesis, humans cannot synthesize AsA by themselves, so they obtain AsA from foods (1).

In higher plants, the L-galactose pathway is a critical metabolic pathway for AsA biosynthesis. At present, the genes involved in this pathway had been identified, cloned and verified from different kiwifruit varieties (2, 3). GDP-D-mannose 3′, 5′-epimerase (GME) catalyzes the conversion of GDP-D-mannose to GDP-L-galactose, and *GME* transcripts were associated with the peak of AsA content in four genotypes of kiwifruit (4). After *GME* expression was silenced, the level of AsA in tomato decreased significantly (5). GDP-L-galactose phosphorylase (GGP) can catalyze the conversion of GDP-L-galactose to L-galactose-1-phosphate. The AsA content of kiwifruit was significantly higher in *Actinidia eriantha* than *Actinidia rufa*, probably because the *GGP* transcripts in *Actinidia eriantha* were significantly higher than *Actinidia rufa* (6). L-galactose dehydrogenase (GalDH) catalyzes the oxidation of L-galactose to L-galactose-1,4-lactone, and L-galactose 1,4-lactone dehydrogenase (GalLDH) is the last enzyme in AsA synthesis. GalLDH catalyzes the conversion of L-galactose 1,4-lactone to AsA and plays a critical role in the accumulation of AsA in plants. In plants, the activity and transcription level of GalLDH were positively correlated with AsA content (7). The accumulation of AsA was closely related to GalLDH activity during fruit development of kiwifruit, and the change trend of GalLDH activity was similar to that of AsA accumulation in kiwifruit (8). Meanwhile, the L-gulose pathway, D-galacturonate pathway and myo-inositol pathway are also involved in AsA synthesis in plants (9–11).

AsA can be regenerated by the AsA-glutathione cycle. Ascorbate peroxidase (APX) and ascorbate oxidase (AO) are used as electron donors to reduce hydrogen peroxide (H_2_O_2_) and superoxide anion (O_2_^–^), respectively, resulting in production of monodehydroascorbate (MDHA). Monodehydroascorbate reductase (MDHAR) and dehydroascorbate reductase (DHAR) can convert MDHA to AsA (9). The reaction of AsA catalyzed by AO was delayed in carrots treated with a pulsed electric field (PEF); therefore, high-energy PEF treatment maintained the stability of AsA (12). In transgenic plants, AsA content was increased by *DHAR* overexpression, indicating that AsA content can be increased by enhancing the AsA cycle (13). *TaMDHAR* is a gene encoding MDHAR, and knock out of *TaMDHAR* in wheat resulted in decreased MDHAR activity and lower AsA content (14).

In plants, melatonin (MT) was first discovered in 1995 (15). Since then, the function and role of MT in plants have been widely studied. For postharvest fruit preservation, many studies have reported that MT has an effect on delaying senescence and is expected to become a new preservative. MT treatment inhibited the expression of the ethylene synthesis genes *ACS* and *ACO* in pear fruits and reduced the ethylene production rate, thereby delayed fruit senescence (16). As a signaling molecule, MT can enhance enzymatic and non-enzymatic antioxidant systems, which can scavenge reactive oxygen species (ROS) in fruits during storage. The non-enzymatic antioxidants included glutathione (GSH), AsA, polyphenols and so on. The AsA content of sweet cherries was increased by MT treatment (17). At present, the effects of exogenous MT treatment on postharvest kiwifruit had been reported. MT could improve the antioxidant capacity of kiwifruit then alleviated chilling injury of the fruit (18). MT also inhibited the respiration rate and ethylene production of kiwifruit and delayed fruit senescence, meanwhile the content of ethanol and acetaldehyde were reduced in the later storage of fruit (19). In addition, exogenous MT treatment significantly increased the AsA content of kiwifruit during storage, but the specific mechanism is not very clear currently. Therefore, the effects of exogenous MT on the AsA content, AsA synthesis and transcription level of cyclic transformation genes in kiwifruit during postharvest storage were studied, which will provide a basis for the regulation of storage quality in postharvest kiwifruit.

## Materials and methods

### Plant material and treatment

‘Jinyan’ kiwifruit (*Actinidia chinensis* Planch) were harvested from Kiwifruit Germplasm Resource Nursery, Fengxin County, Jiangxi Province (28.68° N, 115.31° E) and transported to Jiangxi Key Laboratory for Postharvest Technology and Non-destructive Testing of Fruits & Vegetables on 27 October 2021. The orchard is a commercial orchard belonging to the subtropical humid climate, sandy red soil, pH 5.5–6.5. ‘Jinyan’ kiwifruit are 6-year-old vines which grafted onto rootstock of *A. arguta* (Siebold et Zucc.) Planch. ex Miq. The soluble solids content (SSC) of kiwifruit were about 8% at harvest. After 1 day (d) of sweating, fruits with moderate size, consistent maturity, no infection by pests and mechanical damage were selected for further experiments.

According to our previous experiments (data not published), the fruits were dipped in 0.1 mmol/L MT for 10 min, and distilled water as a control. Both the control and MT treatments were repeated three times, with 200 fruits per replicate. Then, the fruits were dried naturally and placed at 20 ± 1°C and 85–90% relative humidity. During kiwifruit storage, 15 fruits per treatment were selected randomly to determine fruit firmness, SSC, respiration rate and ethylene production rate every 2 days. The remaining samples were frozen and placed at −80°C for further measurement.

### Determination of firmness and soluble solids content

After removing the peel, two symmetrical points on the equatorial region of each fruit were randomly selected to measure fruit firmness by a TMS-Touch texture analyzer, the probe diameter was 8 mm, penetrated the fruits at a speed of 5 mm s^–1^ to a depth of 10 mm, 15 fruits were measured for each treatment, and the results were expressed in newton (N). SSC was determined by PAL-1 digital refractometer and repeated three times per treatment; the results were expressed as %.

### Determination of respiration rate and ethylene production

The respiration rate was measured by a GXH-3051H infrared carbon dioxide fruit and vegetable respiration tester (Jun-Fang-Li-Hua Technology-Research Institute, Beijing, China) with 1,040 μL/L CO_2_ as the calibration standard. The carrier gas was CO_2_-removed air, the gas flow rate was 0.5 L/min, and the respiration rate was calculated as mg CO_2_⋅kg^–1^⋅h^–1^ of five fruits per replicate for a total of three replicates.

For ethylene production measurement, nine fruits per replicate were placed in a 0.8 L closed gas collector for 2 h, and 1 mL of top air was extracted from each container and injected into a Gas Chromatography (GC-2014, Shimadzu, Japan) through a rubber diaphragm and column (DANI, Italy). Detector temperature was 200°C, column temperature was 130°C, current velocity of N_2_ was 5.0 mL min^–1^. The ethylene production was calculated as ng⋅kg^–1^⋅s^–1^.

### Determination of titratable acid and ascorbic acid

For TA analysis, 10 g kiwifruit was grinded to ground and diluted with 20 mL distilled water in a triangular bottle, bathed in 80°C water for 30 min. After cooling and centrifugation, supernatant was used by titration with 0.1 M sodium hydroxide and TA was expressed in % (20). The AsA content was measured as previously reported with a slight modification (21), 3 g of fruits tissue was grinded with 10 mL 0.05 mol L^–1^ oxalic acid-EDTA, the centrifuged at 9,000 × g for 40 min and the supernatant was collected. Then, 2 mL supernatant was mixed with 3 mL oxalic acid-EDTA, 0.5 mL of metaphosphoric acid-acetic acid, and 1 mL of 5% H_2_SO_4_. The mixture was watered at 80°C for 15 min then cooled to room temperature, and the absorbance was measured at 760 nm using AsA as standard. The AsA content was showed as mg⋅100 g^–1^, and the experiment was repeated three times.

### Real-time quantitative PCR

RNA extraction was performed using an RNA extraction kit (TIANGEN). The extracted RNA integrity was tested by 1% agarose gel electrophoresis, and the RNA concentration was determined by evaluating the A260/A280 ratio with a UV spectrophotometer. cDNA was obtained using a YEASEN reverse transcription kit (YEASEN Biotech Co., Ltd.) strictly following the manufacturer’s instructions. Gene expression analysis was performed using a CFX96TM Real-Time System (Bio-Rad, USA) and a Takara kit (TB Green^®^ Premix Ex TaqTM). The total qRT-PCR reaction system was 10 μl, which included 0.3 μl positive and negative gene-specific primers respectively, 5.0 μl TB Green and 3.4 μl ddH_2_O and 1.0 μl cDNA. The relative expression of the gene was normalized to *actin (EF063572*) and expressed as 2^–ΔΔCt^ according to previous methods (22, 23), and the reaction was repeated three times.

### Statistical analysis

The experiment was conducted using a completely randomized design. Excel 2016 was used for data statistics, figures were created by GraphPad Prism 8.0 software, and SPSS 26.0 statistical software was used to analyze the significant differences between the control and MT treatments by two-way ANOVA at *p* < 0.05 (*) or *p* < 0.01 (^**^).

## Results

### Effect of melatonin treatment on firmness and soluble solids content

**FIGURE 1:**
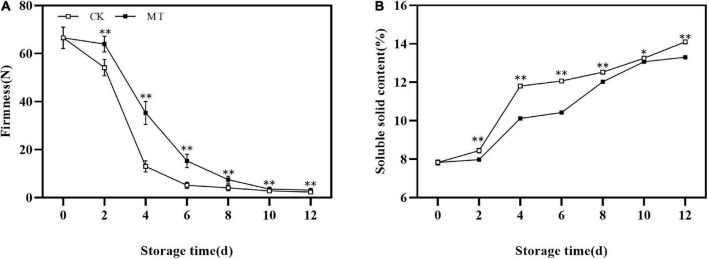
Effects of melatonin (MT) treatment on the firmness **(A)** and soluble solids content (SSC) **(B)** of ‘Jinyan’ kiwifruit. Data are presented as the means ± SE (*n* = 3). Asterisks indicate significant differences between the control and MT treatments (*P < 0.05; ^**^P < 0.01).

The fruit firmness of the two groups decreased continuously under normal temperature storage. MT treatment significantly delayed the decrease in fruit firmness, and there were extremely significant differences between MT treatment and control fruits at 2–8 days of storage (Figure 1A). Meanwhile, the SSC increased in the two groups at all times, and MT delayed the increase of SSC (Figure 1B).

### Effects of melatonin treatment on respiratory rate and ethylene production

**FIGURE 2:**
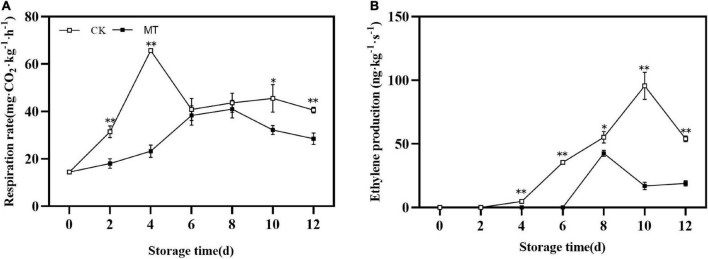
Effects of melatonin (MT) treatment on respiration rate **(A)** and ethylene production **(B)** of ‘Jinyan’ kiwifruit. Data are presented as the means ± SE (*n* = 3). Asterisks indicate significant differences between the control and MT treatments (*P < 0.05; ^**^P < 0.01).

During storage, the changes in the respiration rate and ethylene production of fruits were similar, first increased and then decreased with obvious peaks. MT delayed and inhibited the peak of respiration rate and ethylene production, the peak values were only 66.65 and 52.24% of those in the control group, respectively (Figure 2).

### Effects of melatonin treatment on titratable acid and ascorbic acid

**FIGURE 3:**
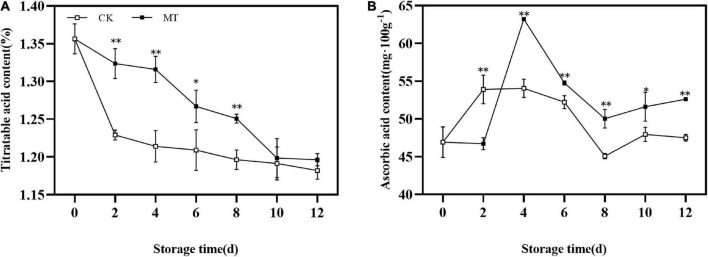
Effects of melatonin (MT) treatment on the titratable acid (TA) **(A)** and ascorbic acid content **(B)** of ‘Jinyan’ kiwifruit. Data represent the means ± SE (*n* = 3). Asterisks indicate significant differences between the control and MT treatments (*P < 0.05; ^**^P < 0.01).

During the whole storage process, the TA content of kiwifruit showed a decreasing trend. MT treatment delayed the decrease in TA of fruits (Figure 3A). The AsA content in the fruits first increased and then decreased, with the peak of MT treatment group appeared later than that of the control group and the peak value of the MT treatment group was 1.17 times that of the control group. At the same time, the AsA content of the treatment group was significantly higher than that of the control group during storage except at 2 days (Figure 3B).

### Effect of melatonin treatment on gene expression of ascorbic acid biosynthesis

**FIGURE 4:**
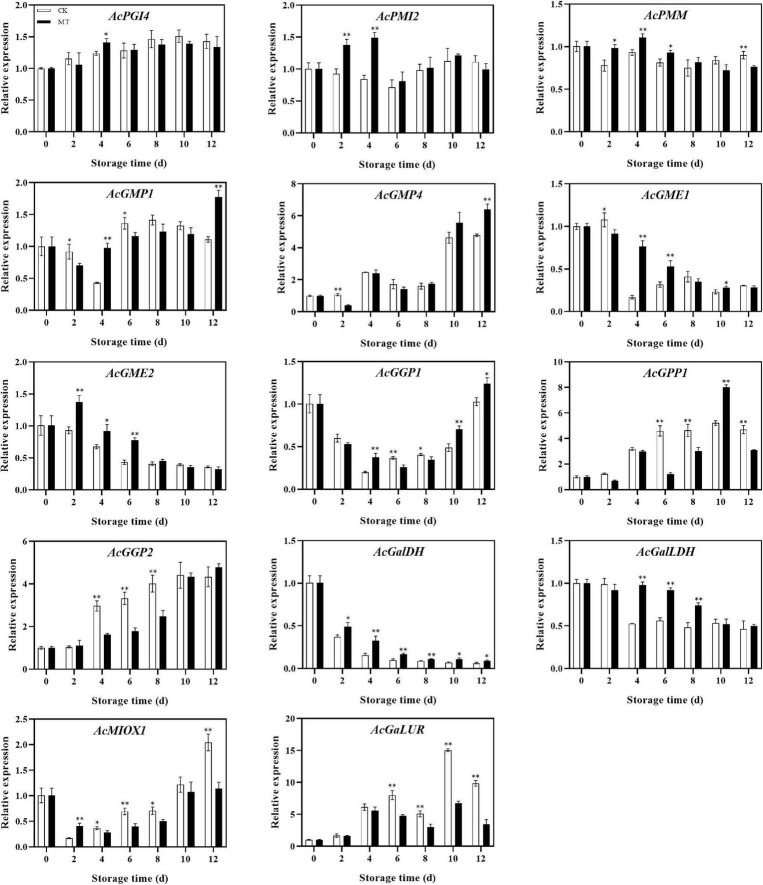
Effects of melatonin (MT) treatment on expression levels of genes involved in ascorbic acid biosynthesis of ‘Jinyan’ kiwifruit. Data represent the means ± SE (*n* = 3). Asterisks indicate significant differences between the control and MT treatments (*P < 0.05; **P < 0.01).

The genes involved in the AsA synthesis process showed different trends. The expression levels of *AcGME2*, *AcGalDH*, and *AcGalLDH* in the MT treatment group were significantly higher than those in the control group, while the expression levels of *AcPMI2*, *AcPMM*, *AcGMP1*, *AcGME1*, and *AcGGP1* were also higher in the MT treatment group at some time points. There was no significant difference in *AcPGI4* or *AcGMP4* expression between the two groups, but the expression levels of *AcGPP1*, *AcGPP2*, *AcMIOX1*, and *AcGalUR*, genes in the inositol pathway and D-galactose aldehyde pathway, were lower in the MT treatment group than in the control group (Figure 4).

### Effect of melatonin treatment on gene expression of ascorbic acid recycling and degradation

**FIGURE 5:**
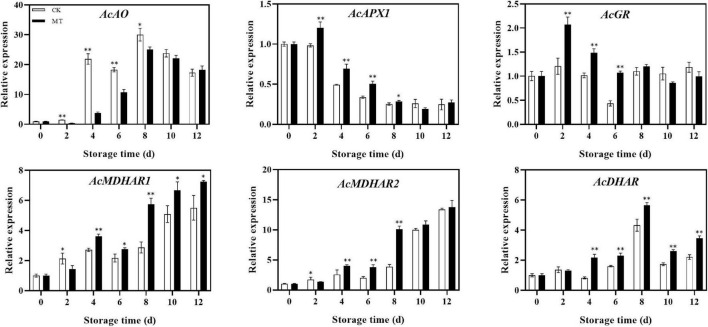
Effects of melatonin (MT) treatment on expression of genes involved in ascorbic acid recycling and degradation of ‘Jinyan’ kiwifruit. Data represent the means ± SE (*n* = 3). Asterisks indicate significant differences between the control and MT treatments (*P < 0.05; **P < 0.01).

MT treatment upregulated the expression of recycling genes, including *AcGR*, *AcMDHAR1*, *AcMDHAR2*, and *AcDHAR*. The expression levels of *AcMDHAR1* and *AcDHAR* were significantly higher in the MT treatment group than in the control group except at 2 days of storage, and the expression levels gradually increased in both groups. In regards to degradation genes, MT treatment upregulated *AcAPX1* expression but downregulated *AcAO* expression. The expression levels of *AcAO* in the MT treatment group were only 17.43 and 58.24% of those of the control group at 4 and 6 days of storage, respectively (Figure 5).

## Discussion

The application of MT on peach and kiwifruit could delay the decline in fruit firmness and SSC (19, 24). The main reason was that MT inhibited the expression of ethylene biosynthesis genes, then the respiration rate and ethylene production were suppressed in fruit (19). Similar results were obtained in this study. As an important antioxidant in plants, AsA participates in the defense of plants against oxidative stress and affects the stability of the cell membrane. MT treatment can increase antioxidant capacity and the AsA content in postharvest fruits, which was confirmed in kiwifruit and pear (18, 25). In this study, MT treatment increased the AsA content of kiwifruit during postharvest storage and improved the antioxidant capacity of kiwifruit, and maintain the stability of membrane lipids, thereby delaying fruit senescence.

AsA accumulation was regulated by L-galactose pathway-related genes. *GME* and *GGP* coexpression significantly increased the AsA concentration in Arabidopsis (26). The transcriptional trends of *GME* and *GGP* were similar during kiwifruit (*Actinidia eriantha*) development and had a significant correlation with AsA levels (8). However, the expression level of *GGP* first decreased and then increased in this experiment. The transcriptional expression level was the lowest when the AsA content reached the peak, which may be related to the feedback regulation mechanism of AsA. Laing et al. (27) reported the feedback regulation mechanism of AsA in *Arabidopsis thaliana*. When the AsA concentration was high, uORFs began to express and inhibit the translation of *GGP*, and *GGP* translation was normal at lower AsA contents. Meanwhile, the expression of *GPP* was negatively correlated with AsA content. This is similar to a study in peach and kiwifruit (28, 29). The expression of *GPP* in the L-galactose pathway is not consistent with AsA accumulation. The correlation between *GPP* and AsA content was also different in various varieties of kiwifruit. The expression level of *GPP* was basically consistent with the AsA content in kiwifruit (*Actinidia deliciosa*), while the correlation between *GPP* expression and AsA content was low in kiwifruit (*Actinidia eriantha*) (6, 8). In this experiment, MT treatment enhanced the expression levels of *AcGME2*, *AcGaLDH*, and *AcGalLDH*, which could be one of reasons for higher AsA content in MT-treated fruits. However, the finding that some synthetic genes were negatively correlated with AsA content needs to be further explored.

In this experiment, the expression levels of *AcMIOX* and *AcGaLUR*, two key genes in the D-galacturonate pathway and myo-inositol pathway, increased during the late storage period and were inhibited by MT treatment at most stages of storage. *GaLUR* expression was significantly higher in the 1-Methylcyclopropene (1-MCP) treatment group than in the control group in the regulation of the AsA metabolic pathway by 1-MCP (29). However, D-galacturonic acid was found to be released during pectin hydrolysis in MT-treated strawberry. MT could maintain fruit firmness and cell integrity, resulting in decreased release of D-galacturonic acid from the cell wall and ultimately affecting AsA synthesis in strawberry (30), which may be the result of lower expression of *AcGaLUR* in kiwifruit treated with MT. The expression level of *MIOX* in kiwifruit (*Actinidia eriantha*) was not significantly correlated with AsA content, indicating that the inositol pathway may had little effect on AsA accumulation in kiwifruit (*Actinidia eriantha*) (8). The specific synthesis of the myo-inositol pathway in kiwifruit still needs further study.

Compared to AsA biosynthesis, the recycling and degradation pathway of AsA may be more important in maintaining AsA content in postharvest fruits. The expression levels of *AcDHAR* and *AcMDHAR1* were upregulated during storage, and MT treatment significantly promoted their expression, which may be the reason for the higher level of AsA content in MT-treated kiwifruits at the later stage of storage. Exogenous MT has also been reported to improve the enzyme activities of *DHAR* and *MDHAR* in kiwifruit leaves, thereby delaying their senescence (31). The previous studies showed that the AsA content was increased by 1.4- and 1.9-fold in transgenic Arabidopsis and tomato with *DHAR* overexpression, respectively, and overexpression of the *MDHAR* gene increased the AsA content by nearly twofold in transgenic tobacco (32–34). For the key degradation gene of AsA, *AO*, we obtained a similar result to that of a previous report (29). MT treatment downregulated the expression level of *AcAO*, and the effect was remarkable in the middle storage period. *Co*mpared with AsA biosynthesis genes, the expression level of *AcAO* gene had a higher fold difference. Therefore, the AsA degradation pathway may play a more important role than the AsA biosynthesis pathway on maintaining the AsA content of postharvest fruits. At the same time, the increase in *AcAO* gene expression was the reason for the gradual decrease in AsA during kiwifruit storage. In this experiment, the expression of *AcAPX1* and *AcGR* was stimulated by MT treatment. MT inhibited the accumulation of O_2_^–^, H_2_O_2_, and malondialdehyde (MDA) and maintained relative membrane permeability by increasing the activities of superoxide dismutase (SOD), catalase (CAT), APX, glutathione reductase (GR), and lipoxygenase (LOX) (19, 21). APX and GR can scavenge reactive oxygen species in fruits, thereby maintaining the stability of the fruit cell membrane. Exogenous MT could increase the content of endogenous MT in fruits (35), and endogenous MT, as a free radical scavenger, also plays a positive role in the antioxidant capacity of fruits, which may be a major reason for the function of MT in delaying fruit senescence during storage. At the same time, whether there was a positive correlation between endogenous MT and AsA content in fruit remains to be further investigated.

## Conclusion

In summary, AsA content in fruits are regulated by multiple pathways and genes. The high expression level of key genes in the L-galactose pathway at the early stage and circulating transformation genes at the later stage maintained the AsA content in postharvest kiwifruits. Meanwhile, MT treatment promoted the expression level of biosynthesis and regeneration genes. It inhibited the expression level of degradation genes. Therefore, MT treatment maintained a higher AsA content and delayed postharvest fruit senescence during kiwifruit storage. However, the synergistic effect of different pathway genes on AsA metabolism requires further study.

## Data availability statement

The original contributions presented in this study are included in the article/Supplementary material, further inquiries can be directed to the corresponding author.

## Author contributions

ZL: investigation, methodology, formal analysis, data curation, and writing—original draft. JZh: investigation and methodology. MX: conceptualization and validation. JZe: formal analysis and data curation. JC: supervision and project administration. MC: conceptualization, writing—review and editing, project administration, and funding acquisition. All authors contributed to the article and approved the submitted version.
