# Supplementary material for: Exogenous melatonin treatment affects ascorbic acid metabolism in postharvest ‘Jinyan’ kiwifruit
Source: Front Nutr. 2022 Dec 2;9:1081476. doi: 10.3389/fnut.2022.1081476 (PMC9755600; doi:10.3389/fnut.2022.1081476)
Supplement: Supplementary file 1 [file Table_1.DOCX]

Supplementary Material

**Table S1**. Primers for real-time quantitative PCR analysis.

| Gene | Forward primer (5’-3’) | Reverse primer (5’-3’) |
| --- | --- | --- |
| *Actin* | TGCATGAGCGATCAAGTTTCAAG | TGTCCCATGTCTGGTTGATGACT |
| *AcPMM* | AAAATGGAAGCAAGGAAGCC | CGAAAATGCGTAATCGTAGTCA |
| *AcPMI2* | TTCACCGAACTCATGTCTGCTAG | CTTATCCGTCAACTGCCTCACC |
| *AcPGI4* | TTATCTGTGACACGGAGCAATG | GCTGTTTATCCGCTCACCATT |
| *AcGPP1* | AGAGGTAATCCGCAAAGGGT | ATCCATGTTGGTTCATCAGTCA |
| *AcGPP2* | ACTGAACCTTTGTGGGATTGC | CGCTGATGTCAAATTCTTTACCG |
| *AcGMP4* | TTGCTGTCATTATGGTCGGTG | TGGCTTGTCCTCTTTCAAGTATC |
| *AcGMP1* | GGTGGATGAGACCGCAACAATC | GGTTGAGTGCCAGCCGATAATG |
| *AcGME1* | TGGAAAGGTGGAAGGGAGAAAGC | ATGAAGGTGAAAGATCGGGTTTGC |
| *AcGME2* | GAGGGGCATTACATCATTGCTTCTG | GGTAAATACAAGCGCTAGAAGCATA |
| *AcGGP1* | GAGGGTGAAAGAGGTTGTTGGTG | CGCAAGCAGTGACATCGTAGC |
| *AcGalDH* | GCTTTGATTTCAGTGCCGAGAGAG | GGGAGTCCTGTAATACCAATAAACCG |
| *AcGalLDH* | CCTGCCAAGGGGACAATAGA | AGGGTTGCACGTCACAACGA |
| *AcMIOX1* | TGTTGCAGACAGCTGAGGCT | CCCACAACAGCCCACTGAGG |
| *AcGaLUR* | GCTCTGAGGTGGGTGTATG | GGTAACGCCTTTATGCTG |
| *AcAO* | AGACTACAGAGCATTCATC | CCATCCATTCCAACTACA |
| *AcAPX1* | AGGGACCCTGGACTGCCAAT | GGCATCCTCATCCGCAGCAT |
| *AcGR* | GTATTCATCCAACATCAG | TTAACTTCAGAGTCCTTC |
| *AcMDHAR1* | ATGAGATGAGAAGAGTTG | TAAGGAAGGTAGTCGTAT |
| *AcMDHAR2* | TATCAAGGGAACTGTGGC | CGGGAATAGAAGTAGGGA |
| *AcDHAR* | TTCTGGTTCTCCTGATGT | ATATTCTTCTCCTCCAAAGTC |
